# Transport and Electrochemical Properties of Li_4_Ti_5_O_12_-Li_2_TiO_3_ and Li_4_Ti_5_O_12_-TiO_2_ Composites

**DOI:** 10.3390/ma15176079

**Published:** 2022-09-01

**Authors:** Anna Kozlova, Nikolai Uvarov, Artem Ulihin

**Affiliations:** 1 Institute of Solid State Chemistry and Mechanochemistry, SB RAS, Kutateladze Str. 18, 630128 Novosibirsk, Russia; 2 Mechanical Engineering Department, Novosibirsk State Technical University, 630071 Novosibrsk, Russia

**Keywords:** Li_4_Ti_5_O_12_-Li_2_TiO_3_, Li_4_Ti_5_O_12_-TiO_2_ composites, solid-state synthesis, ionic conductivity, grain boundary resistance, excess charge capacity

## Abstract

The study demonstrates that the introduction of the electrochemically inactive dielectric additive Li_2_TiO_3_ to LTO results in a strong decrease in the grain boundary resistance of LTO-Li_2_TiO_3_ (LTC) composites at a low concentration of Li_2_TiO_3_. With the increase in the concentration of Li_2_TiO_3_ in LTC composites, the grain boundary resistance goes through a minimum and increases again due to the growth of the insulation layer of small Li_2_TiO_3_ particles around LTO grains. For LTO-TiO_2_ (LTT) composites, a similar effect was observed, albeit not as strong. It was found that LTC composites at low concentration of Li_2_TiO_3_ have unusually high charge–discharge capacity exceeding the theoretical value for pure LTO. This effect is likely to be caused by the occurrence of the electrochemical activity of Li_2_TiO_3_ in the vicinity of the interfaces between LTO and Li_2_TiO_3_. The increase in the capacity may be qualitatively described in terms of the model of two-phase composite in which there is the interface layer with a high capacity. Contrasting with LTC composites, in LTT composites, no capacity enhancement was observed, which was likely due to a noticeable difference in crystal structures of LTO and TiO_2_ preventing the formation of coherent interfaces.

## 1. Introduction

Lithium titanate batteries (LTBs), which use lithium titanate Li_4_Ti_5_O_12_ (LTO) as the anode material, open up new possibilities for the energy storage of lithium-ion batteries (LIBs) with a number of economical as well as ecological aspects. These batteries are characterized by relatively fast recharge, low internal resistance, high charge and discharge rates, long cycle life, high reliability and safety. At present, Toshiba, YABO and Altair Nanotechnologies produce LTBs for electric vehicles and energy storage. Research on LTO-based materials began about 30 years ago to replace graphite as the negative electrode in LIBs [1,2]. LTO is already known to be crystallized in cubic syngony (space group Fd-3m) and have AB_2_O_4_ spinel structure presented as Li[Li_1/6_Ti_5/6_]_2_O_4_. The volume change of the unit cell associated with insertion/extraction of lithium in/out Li_4 + x_Ti_5_O_12_ (0 < x < 3) is very small (~0.2%). Therefore, Li_4_Ti_5_O_12_ is regarded as a zero-strain material for lithium insertion that offers high stability during cycling (up to 10,000 cycles) without significant loss of capacity [3,4,5]. High potential plateau around 1.55 V (vs. Li^+^/Li°), exceeds the reduction potential of most electrolyte solvents, and thus avoids the formation of lithium dendrites on the anode surface. This ensures the safety of LIB with an LTO anode, in contrast to LIB with a graphite anode [4,5,6,7]. However, the main limitation for the wide practical use of LTO is poor electronic and ionic conductivity, which, according to various reports, ranges from 10^−13^ to 10^−7^ S cm^−1^ [8,9,10].

Heterogeneous doping is one of the most promising approaches to materials modification. This approach makes it possible to obtain composite solid electrolytes with high ionic conductivity [11,12,13,14]. The introduction of heterogeneous additives can lead not only to improved charge-discharge characteristics, but also to an increase in the specific capacity of the electrode material. As Krajewski et al. (2016) reported, CV and EIS measurements show an increase in Li^+^ chemical diffusion coefficient values with increasing the amount of Ag nanoparticles in the LTO powder up to 3% wt. [15]. Galvanostatic charge/discharge studies (Krajewski et al., 2017) demonstrate the enhanced electrochemical properties of Li_4_Ti_5_O_12_ powder after its modification with Ag–Cu nanoparticles mixture, namely ca. 12.5% increase in specific capacity retention and ca. 19 mAh g^−1^ increases in specific capacity at a current rate of 10 C were observed [16]. The authors suggest the reason of improved electrochemical properties to be surface activation through contact with conductive nanoparticles, which leads to an increase in the electrochemically active electrode surface as well as in the reaction rate. Dielectric phases can also be used as heterogeneous additives for doping electrode materials. It was recently discovered that LTO-based composite electrode materials with additives of the electrochemically inert phase Li_2_TiO_3_ have better electrochemical characteristics compared to pure LTO-based anode material [17,18].

The phase β-Li_2_TiO_3_ with monoclinic structure (space group C2/c) is the most studied modification of Li_2_TiO_3_ [19,20]. The values of the specific discharge capacity of β-Li_2_TiO_3_ depend on the particle size or the discharge rate. For micrometer-sized Li_2_TiO_3_ particles, the discharge capacity is about 12–15 mAh g^−1^ for the first five discharge cycles [21,22]. The specific discharge capacity of nanofibers on the first cycle is 18.77 mAh g^−1^ [21]. Large capacity loss on the first cycle and less strong but continuous decrease in capacity on the next 10 cycles indicates that Li_2_TiO_3_ is electrochemically inactive, and its reversible capacity can be neglected.

Nevertheless, the addition of Li_2_TiO_3_ as a structurally compatible dopant to LTO [17,18] demonstrates not only a better rate capability but also higher specific discharge capacities (216 mAh g^−1^ at 0.1 C and 194 mAh g^−1^ at 0.5 C) as compared to LTO (175 mAh g^−1^) [23]. As Li_2_TiO_3_ is an electrochemically inert material, the authors (Li et al., 2017; Bhatti et al., 2016) suggest that Li_2_TiO_3_ inclusions could improve the lithium ion conductivity of the composite nanofibers and, in addition, enable intercalating of more lithium ions to the interfaces of the composite nanofibers which can be considered as additional lithium storage sites [23,24].

In this study, LTO-Li_2_TiO_3_ and LTO-TiO_2_ composites have been chosen as systems for studying the electrophysical and electrochemical properties. Both composites were obtained by solid-phase synthesis by changing the Li/Ti ratio. In the case of Li excess, the Li_2_TiO_3_ phase is formed as an additional phase, and in the case of Ti excess the composites contain TiO_2_ phase.

## 2. Materials and Methods

### 2.1. Synthesis

The samples of LTO, LTO-Li_2_TiO_3_ and LTO-TiO_2_ composites were synthesized by solid-state reaction. As the initial reagents, Li_2_CO_3_ (Chemical grade, Rare Metals Plant Inc., Novosibirsk, Russia) and TiO_2_ (rutile, Chemical grade, Interkhim Inc., Novosibirsk, Russia) powders were used. For the synthesis of LTO, LTO-TiO_2_ and LTO-Li_2_TiO_3_ composites the initial reagents were taken in amount corresponding to different atomic ratio to Li:Ti. The final products of synthesis obtained at the Li:Ti ratios equal to 2.8:5, 3.2:5, 3.6:5, 4:5, 4.2:5, 4.4:5, 4.6:5, 4.8:5 and 5.2:5 were denoted as LTT-3, LTT-2, LTT-1, LTO, LTC-05, LTC-1, LTC-15, LTC-2 and LTC-3, respectively. The initial reagents were preliminarily mixed in agate mortar and then mechanically treated in a high-energy planetary mill AGO-2 in steel jars by steel balls (diameter 8 mm) for 5 min at a rate of 400 rpm. Prior milling of a small portion of the mixture was milled in order to line surfaces of the jar and the balls with the treated material in order to avoid possible contamination of the treatment products by the wearing material from the jar and balls. After treatment, the mixtures were pressed into pellets and annealed at 950 °C for 5 h.

### 2.2. Characterization

Phase identification of the prepared samples was carried out by X-ray diffraction (XRD) using a D8 Advance powder diffractometer (Bruker) in Θ-Θ -geometry under Bragg–Brentano focusing with CuK_α_ radiation source and a one-dimensional Lynx-Eye detector with a nickel filter. The ICDD-PDF2 database was used for phase identification. Quantitative phase analysis and refinement of the structure parameters were performed by the Rietveld method using the Topas 4.2 program.

The morphology of the LTC samples was studied by high-resolution transmission electron microscopy (HREM) on a dual-corrected transmission electron microscope Themis Z (Thermo Fisher Scientific) with an accelerating voltage of 200 kV and a limiting resolution of 0.07 nm and 0.06 nm.

To study the transport properties and ionic conductivity, the test samples were compressed into the pellets at 20 MPa and sintered at 950 °C for 5 h in air. Silver paste electrodes were deposited on the surfaces of the pellets. Electrical measurements were carried out in air in the temperature range from 35 to 250 °C in forevacuum in a stepwise isothermal mode using a HP-4284A Precision LCR Meter in the frequency range of 30 Hz–1 MHz.

To identify electrochemical properties, 80 wt.% of active material under study, 6 wt.% of carbon black, 4 wt.% of nanotubes, and 10 wt.% of PVDF binder were mixed and dispersed in N-methylpyrrolidone (NMP) to form slurry. Copper foil 15 μm in thickness was coated with the resulting slurry and dried in a vacuum oven at 120 °C overnight. The total loading of the film coating was approximately 1.5–2 mg cm^−2^. The coated foils were punched into 8 mm diameter disks to obtain the working electrode, and dried at 120 °C overnight under vacuum before being placed in the glovebox for cell assembling. The electrolyte used was a mixture (1:1 by mass) of1 M LiPF_6_ solution in ethylene carbonate (EC) and dimethylcarbonate (DMC). The electrodes were assembled inside a dry glovebox chamber into coin cell of the CR2032 type with the metallic lithium counter electrode. Charge/discharge curves were recorded on ACK 2.5.10.1 analyzer in the galvanostatic mode at various current densities between 1.0 and 2.5 V (vs. Li^+^/Li°).

## 3. Results

### 3.1. Structural Studies

**Figure 1:**
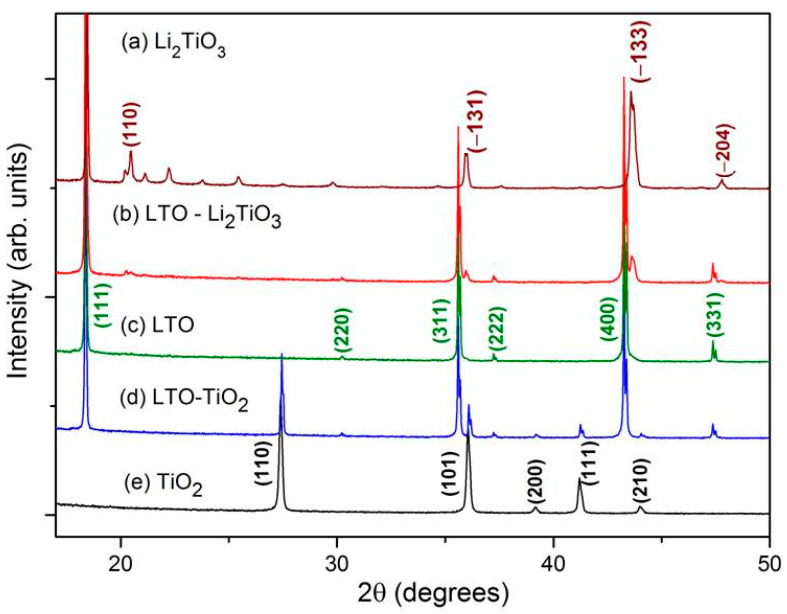
XRD patterns of samples synthesized at different ratios of Li:Ti in the initial mixtures: composite (b) LTC-2 (Li:Ti = 4.8:5), (c) single phase LTO (Li:Ti = 4.0:5) and (d) LTT-2 composite (Li:Ti = 3.2:5) in comparison with the patterns of pure phases (a) Li_2_TiO_3_ and (e) TiO_2_ (rutile).

Figure 1 shows the XRD patterns of LTO, LTC-2 and LTT-2 composites, corresponding to the two-phase systems Li_4_Ti_5_O_12_-Li_2_TiO_3_ and Li_4_Ti_5_O_12_-TiO_2_, respectively. The content of the impurity phases β-Li_2_TiO_3_ or TiO_2_ depends on the relative amount of lithium in the initial mixture. The excess Li content (atomic ratio of Li/Ti > 4/5) produces a two-phase system Li_4_Ti_5_O_12_-Li_2_TiO_3_ as shown in Figure 1 b whose patterns include overlapping XRD peaks of monoclinic β-Li_2_TiO_3_ (ICDD-33-0831) Figure 1 a and cubic Li_4_Ti_5_O_12_ (ICDD-49-0207) Figure 1 c. If lithium is deficient (atomic ratio of Li/Ti < 4/5), two-phase systems Li_4_Ti_5_O_12_-TiO_2_ as shown in Figure 1 d are formed when the impurity phase is rutile TiO_2_ with orthorhombic structure (ICDD-21-1276) (Figure 1e).

The Li_2_TiO_3_ and TiO_2_ phases concentrations in the composites determined from the XRD profiles were 4.6; 9.7; 16.4; 19.7 and 28.4 wt.% for LTC-05, LTC-1, LTC-15, LTC-2 and LTC-3 composites and 11.6 and 20.3 wt.% for LTT-1 and LTT-2 composites, respectively. These values correlate well with the calculated data presented in Table 1.

**Figure 2:**
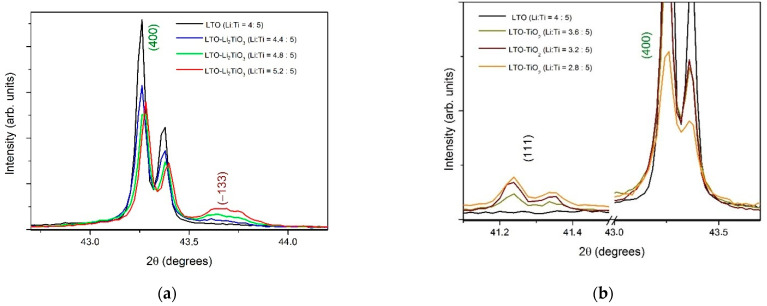
The sections of XRD patterns showing both the reflections of LTO and the impurity phase in the pure LTO sample and (**a**) in LTC-1, LTC-2, LTC-3 and (**b**) LTT-1, LTT-2, LTT-3 composites.

Thus, composites with a certain percentage of the additional phase can be obtained by changing the deviation from the stoichiometric ratio Li:Ti = 4:5. Figure 2 shows a detailed part of the XRD patterns in the 2θ range from 41 to 44°. It is clearly seen that by increasing or decreasing the Li content in the initial reagents, it is possible to obtain the final product with a given content of impurity phase of a certain type, which is confirmed by calculations performed by the Rietveld method.

### 3.2. Ionic Conductivity

**Figure 3:**
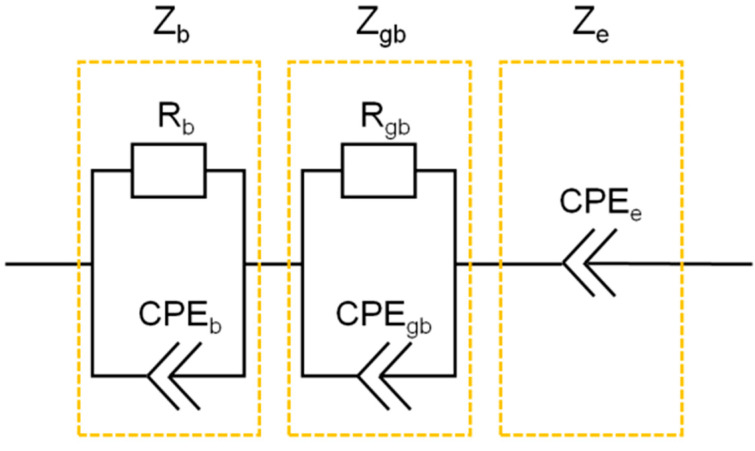
The equivalent circuit used to interpret the results of electrical measurements.

The electrical properties of the synthesized materials were studied through the method of impedance spectroscopy. For the analysis of the impedance spectra at the temperature range from 35 to 250 °C the equivalent circuit was proposed as shown in Figure 3.

The circuit includes three impedances connected in series related to the impedance of the bulk material, Z_b_, the grain boundary impedance Z_gb_, and the electrode impedance Z_e_. Each element of the circuit corresponds to a different stage of ion transport or polarization in the material. Bulk impedance and grain boundary impedances include two elements: resistance (R_b_ and R_gb_) and constant phase element (*CPE_b_* and *CPE_gb_*). The electrode impedance is described by the constant angle element *CPE_e_*, a special case of which is the Warburg impedance. The volume resistance of the sample is related to the value of the volume conductivity, *σ_b_*, by the ratio *σ_b_* = 1/R_b_ (d/S), where *d* is the pellet thickness and *S* is the electrode area. The value of the bulk conductivity is described by the Arrhenius equation:

(1) $$\sigma_{b}=\frac{A_{b}}{T}\cdot \exp\left(-\frac{E_{b}}{kT}\right)$$

The grain boundary resistance R_gb_ can be represented as *σ_gb_* = 1/R_gb_·(d/S), where *σ_gb_* is a parameter which has the dimension of conductivity and also obeys the Arrhenius dependence:

(2) $$\sigma_{gb}=\frac{A_{gb}}{T}\cdot \exp\left(-\frac{E_{gb}}{kT}\right)$$

The *CPE_b_*, *CPE_gb_*, and *CPE_e_* values are defined by the equations

(3) $$CPE_{b}=Y_{b}\cdot {\left(i\omega \right)}^{\alpha_{b}}$$

(4) $$CPE_{gb}=Y_{gb}\cdot {\left(i\omega \right)}^{\alpha_{gb}}$$

(5) $$CPE_{e}=Y_{e}\cdot {\left(i\omega \right)}^{\alpha_{e}}$$

where *Y_b_*, *Y_gb_* and *Y_e_* are constants; *α_b_*, *α_gb_* and *α_e_* are the exponents (0 < *α_b_*, *α_gb_*, *α_e_* < 1) and ω is the angular A.C. frequency. Empirically, it was found that the best agreement between theoretical and experimental data is observed assuming that *CRE_b_* values depend on temperature according to the expression:

(6) $$Y_{gb}=\frac{Y_{gb}}{T}\cdot \exp\left(-\frac{\left(1-\alpha_{gb}\right)E_{gb}}{kT}\right)$$

The reasons for this dependence remain unclear and require additional theoretical analysis which is beyond the scope of this study. The expression for the Warburg impedance was used as the electrode impedance, i.e., it was assumed that in all cases α_e_ = 0.5.

Thus, 11 independent parameters were used to fit the theoretical dependencies (1–6) to the experimental data in the temperature range 35–250 °C: A_b_, E_b_, ε_b_, Y_b_, α_b_, A_gb_, E_gb_, ε_gb_, Y_gb_, α_gb_, Y_e_. The data obtained at 30 different A.C. frequencies in the frequency range of 30 Hz–1 MHz at 20–30 temperatures, i.e., a total of 600–900 experimental points, were used for the analysis. Fitting was performed using Mathcad 11.0 and UTCMathcad 15.0 software.

**Figure 4:**
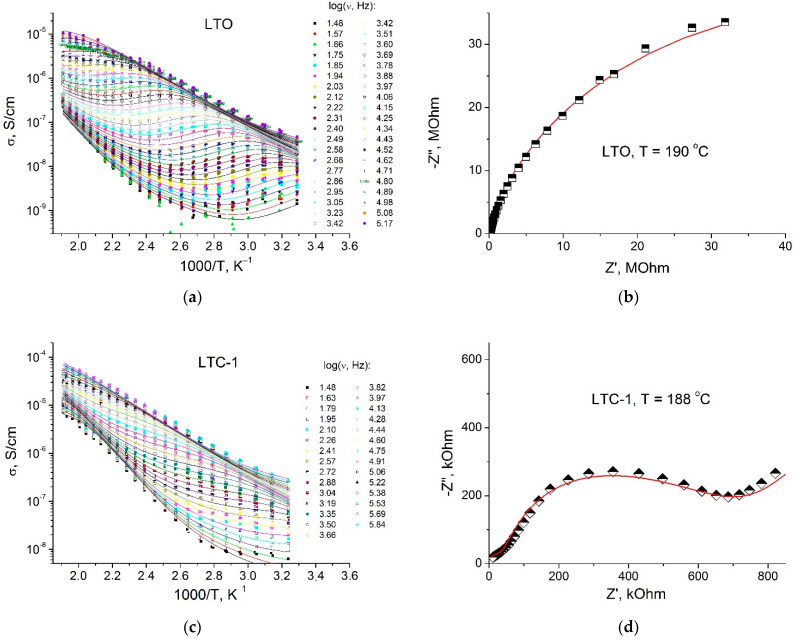

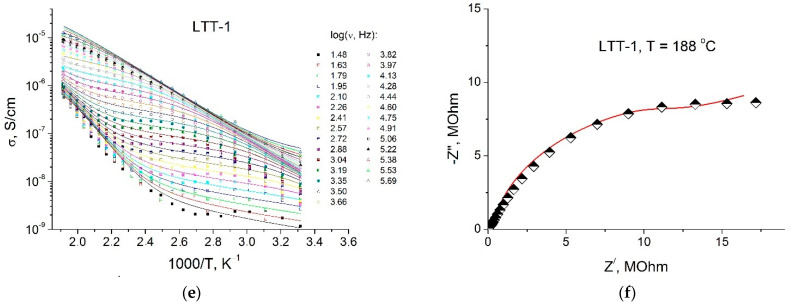
Temperature dependences of the real part of conductivity, measured at different frequencies (indicated in the plots) (**a**,**c**,**e**) and Nyquist plots (**b**,**d**,**f**) obtained at temperatures 188–190 °C for LTO, LTC-1 and LTT-1 samples. Dots are experimental values; lines are theoretical curves obtained as a result of fitting.

Typical fitting results are demonstrated in Figure 4a,c,e for samples LTO, LTC-1 and LTT-1, respectively. Figure 4b,d,f show some Nyquist plots for these samples. The numerical values of the equivalent circuit parameters obtained by fitting are shown in Table 2. The first element of the equivalent circuit, Z_b_, describing the transport of ions through the volume of the material, is characterized by the volume conductivity with the activation energy *E*_b_ = 0.47–0.53 eV. This value of the activation energy agrees well with the calculated data presented in the papers [25,26]. The value of LTO bulk conductivity at room temperature determined in this work (1.8 × 10^–8^ S cm^−1^) is within the range reported in the literature: from 8 × 10^–10^ [27] to 7.6 × 10^–8^ S cm^−1^ [28]. The low conductivity values can be explained by the absence of vacancies in positions 8*a* and interstitial lithium ions in positions 16*c* of the spinel structure. The second element of the equivalent circuit, describing the process of ion transport across grains surfaces, is characterized by the grain boundary resistance R_gb_ with the activation energy *E*_gb_ = 0.90–1.1 eV. This process is limited by the contribution of the grain boundary resistance to the total impedance of the sample. At a high temperature, the electrode polarization effect described by CPE_e_ is observed, indicating a predominantly ionic character of conductivity.

The conductivity measured at direct current (assuming negligibly small electrode impedance contribution), σ_dc_ = (σ_b_^−1^ + σ_gb_^−1^)^−1^, at low temperatures is determined by the value σ_gb_. This value, determined by the grain boundary resistance, depends on the particle size of the samples, the density of the pellet, and the presence of impurities adsorbed on the grain’s surfaces. The addition of a small amount of Li_2_TiO_3_ to the LTO leads to a decrease in grain boundary resistance and an increase in values σ_gb_. Despite the difference in the symmetry of the crystal structures, LTO and Li_2_TiO_3_ compounds have similar chemical and physical characteristics, their densities at room temperature differ by 1.5%. Consequently, the adhesion between LTO and Li_2_TiO_3_ can be expected to be strong, and a good interface contact is formed between the components during sintering of their mixture. In this case, at the LTO/Li_2_TiO_3_ interface, additional point defects may form due to interface interaction accompanied by the transfer of cations from one phase to another. Similar processes are characteristic of composite solid electrolytes [12,13].

As a result, the concentration of charge carriers near the interface increases, which leads to a decrease in the grain boundary resistance (or to an increase in σ_gb_ values) for LTC-1, LTC-2 samples. When the concentration of Li_2_TiO_3_ increases, small particles of the dielectric phase Li_2_TiO_3_ accumulate around LTO particles leading to an increase in the resistance of the grain boundaries and a decrease in the values of σ_gb_. A similar effect is observed in LTT composites, but the relative effect of intergrain resistance reduction in these composites is lower compared to LTC composites.

### 3.3. Electrochemical Properties

**Figure 5:**
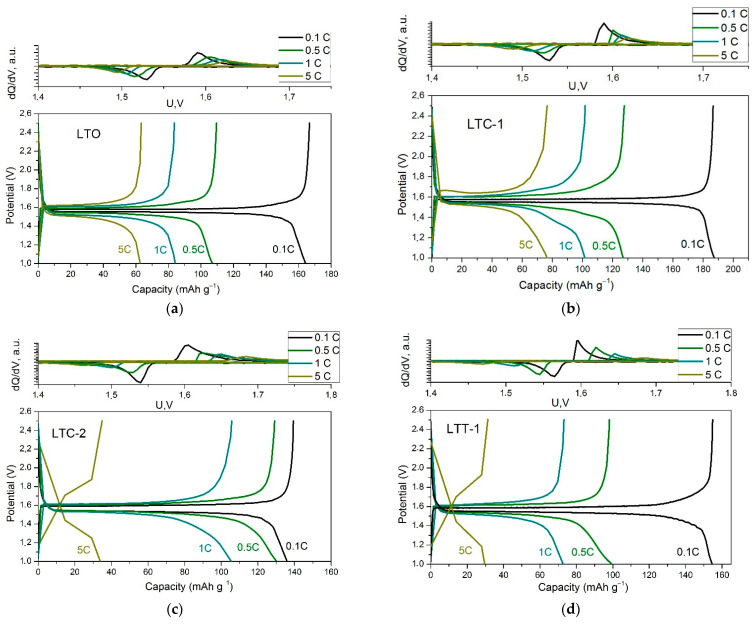
The charge-discharge curves obtained for the studied samples in the region of potentials 1.0–2.5 V (vs. Li^+^/Li°) at the 2-nd cycle at different charge/discharge rates. Corresponding dQ/dU vs. U curves are presented in the upper plot for (**a**) LTO, (**b**) LTC-1, (**c**) LTC-2 and (**d**) LTT-1 samples.

In order to investigate the influence of the Li_2_TiO_3_ and TiO_2_ additives on the electrochemical properties of LTC and LTT composites, charge-discharge experiments were carried out. Figure 5 shows the charge-discharge curves for the samples under study measured at different charge/discharge rates in the potential range 1.0–2.5 V (vs. Li^+^/Li°). Charge-discharge curves for LTO have a plateau at the potential range of 1.55–1.6 V, corresponding to the reversible electrochemical reaction [29]:

Li_4_Ti_5_O_12_ + 3Li^+^ + 3e^−^↔ Li_7_Ti_5_O_12_ (7)

The theoretical value of the electrochemical capacity for this process is 175 mAh g^−1^. The experimental values of average voltage and specific discharge capacity for the LTO sample obtained in this work at a discharge rate of 0.1 C are 1.6 V and 166 mAh g^−1^, respectively. Such values are typical for anode materials based on LTO when measured in the voltage range above 1 V (vs. Li^+^/Li°).

The theoretical value of the change–discharge capacitance of the two-phase composite can be obtained for the case when the electrochemical reaction proceeds independently for each component. In this case one can use the sum rule and obtain a linear relationship for the specific capacity related to the mass of the composite, *Q_th_* measured in [mAh g^−1^]:

(8) $$Q_{th}=\left(1-w\right)Q_{1}^{0}+wQ_{2}^{0}$$

where *w* is the mass fraction of the impurity phase, $Q_{1}^{0}$ and $Q_{2}^{0}$ are the values of the theoretical capacity of individual compounds, LTO and heterogeneous impurity, respectively, in the potential range under study.

The choice of the correct values for the theoretical capacity of $Q_{1}^{0}$ and $Q_{2}^{0}$ depends on the values of the voltage range in which the charge–discharge curves are obtained. When operating in a wider voltage range (below 1 V vs. Li^+^/Li°), the discharge curves for pure LTO show an additional plateau at 0.5 V, leading to higher theoretical capacitance values. In the present work, we do not extend the potential range beyond the voltages below 1 V (vs. Li^+^/Li°), so a value of 175 mAh g^−1^ was taken as the theoretical value of *Q*_1_^0^. The charge-discharge curves for the Li_2_TiO_3_ compound also show a broad plateau in the voltage region of 0.2–1.5 V (vs. Li^+^/Li°), which is observed in the nanostructured samples and is characterized by the theoretical capacity value of 170–200 mAh g^−1^ [30]. However, in the region of voltages above 1 V (vs. Li^+^/Li°), used in the present study, the experimental values of Li_2_TiO_3_ specific capacity obtained at low charge/discharge rates do not exceed 15 mAh g^−1^ [17,18]. It suggests that pure Li_2_TiO_3_ should be electrochemically inert material. In respect to TiO_2_, the ordinary crystalline state of TiO_2_ is known to intercalate only a few amounts of Li. The theoretical value of the capacity of TiO_2_ rutile does not exceed 15 mAh g^−1^ [31,32,33], which is close to the corresponding value for Li_2_TiO_3_. Therefore, in further calculations it was assumed that the theoretical value of the specific capacity of heterogeneous impurity for both Li_2_TiO_3_ and TiO_2_ is approximately equal to $Q_{2}^{0}$ = 15 mAh g^−1^.

**Figure 6:**
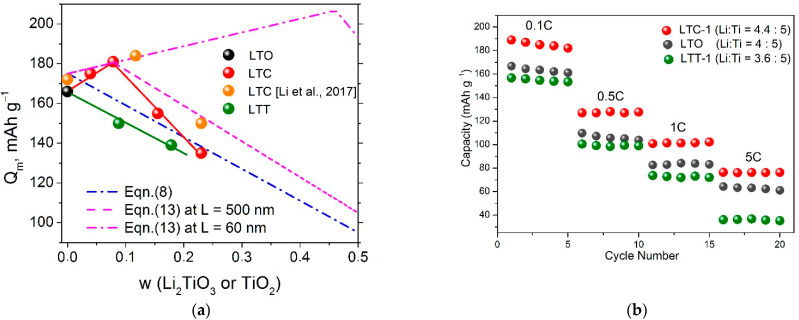
The variation of the discharge capacity as a function of the mass fraction of the additive (**a**) and the discharge rate (**b**) in LTC and LTT composites. Points are experimental values obtained at the rate of 0.1C in the present work (black, magenta and green points for LTO, LTC and LTT composites, respectively) as well as data (orange points) reported earlier for LTC composites in the paper [23]. Red and green lines are guides for eyes to visualise experimental data for LTC and LTT composites. Blue line corresponds to the theoretical dependences obtained from Equation (8) and magenta lines were obtained using Equation (13) for two values of the LTO grain size: L = 500 nm or 60 nm with parameters λ = 6 nm, $Q_{1}^{0}$ = 175 mAh g^−1^, $Q_{2}^{0}$ = 15 mAh g^−1^ and $Q_{S}^{0}$ = 244 mAh g^−1^.

Figure 6a shows the variation of the capacity with the mass fraction of the additives in the LTC and LTT composites in comparison with the theoretical relationship, Equation (8). As seen, the capacitance of composites should monotonically decrease with increasing concentration of the additive, Li_2_TiO_3_ or TiO_2_. Nevertheless, at low concentrations of Li_2_TiO_3_ the capacity of LTO-Li_2_TiO_3_ (LTC) composites markedly increases, reaching a value of 187 mAh g^−1^ which exceeds the theoretical value of the capacity of pure LTO (175 mAh g^−1^). With further increase in the concentration of Li_2_TiO_3_ additive, the capacity of composites LTO-Li_2_TiO_3_ decreases to the values expected from Equation (8). In contrast to LTO-Li_2_TiO_3_ composites, the discharge capacity of LTO-TiO_2_ composites monotonically decreases with increasing TiO_2_ concentration in good agreement with the theoretical dependence (8).

## 4. Discussion

**Figure 7:**
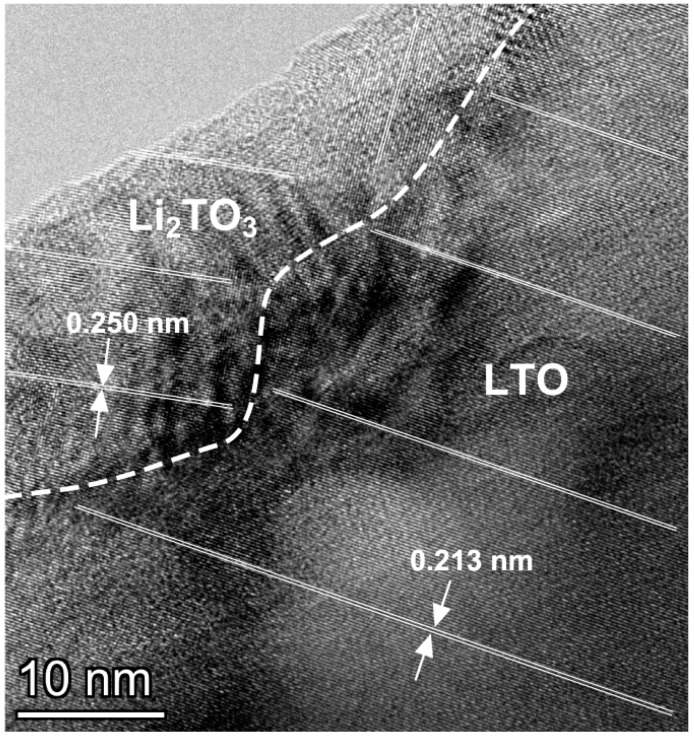
An HRTEM image obtained from the near-surface area of the LTC composite particle. Lines correspond to the crystallographic planes (200) of LTO and (400) or (−313) planes of Li_2_TiO_3_. The interface position is roughly indicated by a dotted line.

Thus, the effect of increasing the specific capacity reported earlier [23,24] is fully confirmed by the results of the present study. This effect is typical only for the LTO-Li_2_TiO_3_ system. The additional contribution of LTO/Li_2_TiO_3_ interfaces provides additional sites for the introduction of lithium cations during electrochemical cycling. The effect can only be explained by a sharp increase in the partial capacitance of Li_2_TiO_3_ in the composite, which is caused by the interface interaction between Li_2_TiO_3_ and LTO and leads to the formation of coherent interfaces (due to a structural similarity) and appearance of the electrochemical activity of Li_2_TiO_3_ in the composite. The grain size (more correctly, the size of the coherent regions) of LTO and Li_2_TiO_3_, determined using the Rietveld full-profile analysis, are 500 and 80 nm, respectively, large particles of LTO are surrounded by smaller Li_2_TiO_3_ grains. High-resolution electron microscopy study showed that in LTC composites Li_2_TiO_3_ phase is located near the surfaces of large LTO crystallites. Figure 7 shows electron microscopy image of the Li_2_TiO_3_/LTO interface. It is seen that crystal lattices of the adjacent phases coherently stacks without visible defects, pores and cracks.

**Figure 8:**
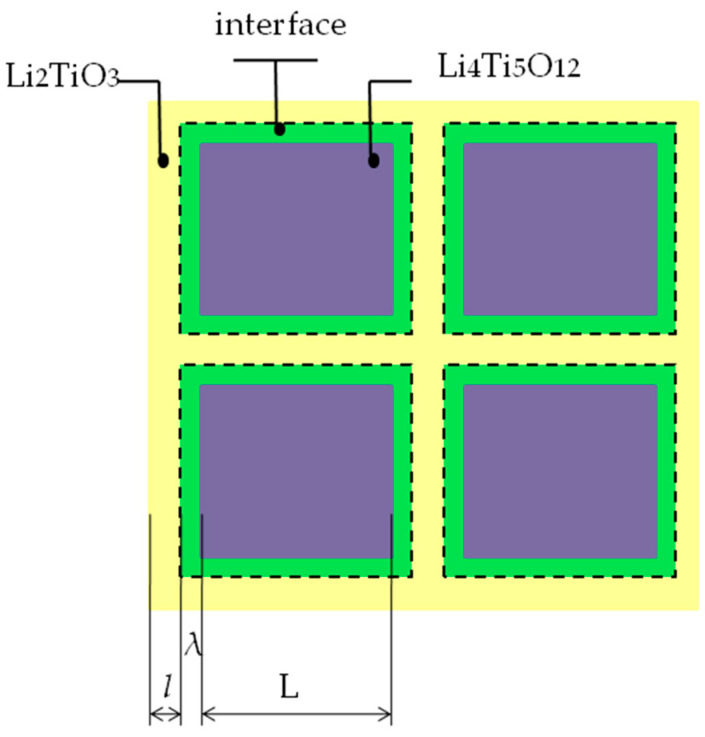
A simplified model of the LTO-Li2TiO3 composite.

Let us assume that the composite consists of cubic LTO particles with the size of *L* covered by a layer of the second phase Li_2_TiO_3_ with the thickness of *l* as shown in Figure 8. In the vicinity of the LTO/Li_2_TiO_3_ interfaces there is a layer of the electrochemically active Li_2_TiO_3_ phase having a higher specific capacity with a characteristic thickness of *λ*. In such approximation, the volume fraction of Li_2_TiO_3_ monotonically increases with the thickness of the Li_2_TiO_3_ phase. When the concentration of Li_2_TiO_3_ is small (at *l* ≤ λ) practically all volume of this phase gets into the interface region. At higher concentrations (at *l* > λ) only part of Li_2_TiO_3_ phase remains electrochemically active.

Then, the volume fractions of LTO, Li_2_TiO_3_ and the interface phase of Li_2_TiO_3_, *f*_1_, *f*_2_ and *f*_S_ can be estimated using dimensionless parameters *α* = λ/*L* and *β* = λ/*L*, respectively, as follows:

(9) $$f_{1}={\left(1-\frac{2\alpha }{1+2\alpha }\right)}^{3}$$

(10) $$f_{2}=1-{\left(1-\frac{2\alpha }{1+2\alpha }\right)}^{3}$$

(11) $$f_{S}=f_{2}\;(\mathrm{at}\;\alpha \;\leq \;\beta );\;f_{S}={\left(\frac{1+2\beta }{1+2\alpha }\right)}^{3}-{\left(\frac{1}{1+2\alpha }\right)}^{3}\;(\mathrm{at}\;\alpha \;>\;\beta )\;$$

The volume fractions of all the phases can be calculated varying the thickness of the Li_2_TiO_3_ phase, *l*, (or the parameter *α*) from zero to infinity at the fixed value of the interface thickness λ (or the parameter *β*). Mass fractions of the phases can be calculated using relations:

(12) $$w_{1}=\frac{\rho_{1}f_{1}}{\rho_{1}f_{1}+\rho_{2}f_{2}};\;w=\frac{\rho_{2}f_{2}}{\rho_{1}f_{1}+\rho_{2}f_{2}};\;w_{S}=\frac{\rho_{2}f_{S}}{\rho_{1}f_{1}+\rho_{2}f_{2}}$$

where *p*_1_ and *p*_2_ are densities of the LTO and Li_2_TiO_3_, respectively; the density of the interface phase is supposed to be similar to one for Li_2_TiO_3_ phase. The theoretical value of the charge-discharge capacity may be represented in the equation:

(13) $$Q_{th}=\left(1-w\right)Q_{1}^{0}+\left(w-w_{S}\right)Q_{2}^{0}+w_{S}Q_{S}^{0}$$

where $Q_{S}^{0}$ is the theoretical value for pure interface phase of Li_2_TiO_3_. Equation (13) differs from Equation (8) by the presence of the additional term *w_S_*$Q_{S}^{0}$, corresponding to the contribution of the interfaces (formally regarded as the interface phase) to the overall capacity of the material. In first approximation as a highest limit for the $Q_{S}^{0}$ value, one can assume that during charge/discharge this interface phase of Li_2_TiO_3_ may be converted into the limiting composition of Li_3_TiO_3_ corresponding to the specific charge/discharge capacity of 244 mAh g^−1^, remaining electrochemically inactive outside the interface region. From the values *L* = 500 nm or 60 nm, *p*_1_ = 3.4 g cm^−3^; *p*_2_ = 4.0 g cm^−3^ and assuming that *λ* = 6 nm and $Q_{S}^{0}$ = 244 mAh g^−1^, one can obtain theoretical dependences *Q_th_* = *f*(*w*), Equation (13), for the LTC composites with maxima qualitatively describing experimental values. It is clear that at low concentrations both theoretical and experimental data exceed the values predicted by Equation (8) and correspond to an upper limit of capacitance for ordinary mixture of the components. Figure 6 also shows that decrease in the grain size of LTO should lead to an increase in the capacitance and the shift of the capacitance maximum to the higher concentration of Li_2_TiO_3_ in the composites. This is the tendency for the composites obtained earlier by electro-spinning method with the LTO grain size of 60 nm [23]. In general, the model provides a rather good fit at low concentrations of Li_2_TiO_3_, whereas at high *w* experimental capacity values decrease, possibly, due to diffusion limitations.

Besides high capacity, LTC composites at low concentration of Li_2_TiO_3_ have a better cycling rate and stability compared to LTO as shown in Figure 6a. This effect is caused by the higher ionic conductivity of the composites, as demonstrated in the impedance measurements above. Due to a strong difference in crystal structures of LTO and TiO_2_, no strong interface interaction takes place in LTT composites. As a result, no additional increase in the electrochemical capacity and much less decrease in the grain boundary resistance is observed in these composites. At high concentration of Li_2_TiO_3_ the grain boundary resistance becomes high again and it leads to the decrease in the capacity as mentioned above.

No effect of the capacity enhancement is observed in LTT composites even at low concentration of the TiO_2_ dopant. The reason appears to be negligible contribution of interfaces to the overall capacity of the composite. This fact may be explained by weak interface interaction between LTO and TiO_2_ due to a strong structure discrepancy between the phases.

## 5. Conclusions

Samples of pure Li_4_Ti_5_O_12_ (LTO) with stoichiometric ratio of Li:Ti = 4:5 and composites LTO-Li_2_TiO_3_ (LTC) and LTO-TiO_2_ (LTT) with excess amount of Li (Li:Ti > 4:5) and deficient in Li (Li:Ti > 4:5), respectively, can be prepared and investigated by X-ray diffraction, impedance spectroscopy and electrochemical charge-discharge techniques. This study shows that introduction of electrochemically inactive dielectric materials Li_2_TiO_3_ and TiO_2_ to lithium titanium oxide LTO results in two non-trivial interrelated effects:

(i) A strong decrease in the grain boundary resistance of LTC composites at low concentration of Li_2_TiO_3_. The effect may be caused by easy formation of coherent interfaces between structurally similar phases Li_2_TiO_3_ and LTO leading to redistribution of lithium ions in the vicinity of the interfaces. With the increase of the concentration of Li_2_TiO_3_ in LTC composites, the grain boundary resistance goes through a minimum and increases again due to growth insulation layer of small Li_2_TiO_3_ particles around LTO grains. For LTT composites, a similar effect was observed, albeit not as strong.
(ii) Unusually high charge–discharge capacity of LTC composites at a low concentration of electrochemically inactive phase Li_2_TiO_3_ exceeding the theoretical value for pure LTO. This effect is likely to be caused by the appearance of the electrochemical activity of Li_2_TiO_3_ in the composite. This effect results from the interface interaction between LTO and Li_2_TiO_3_ taking place due to formation of coherent interfaces between structurally similar phases. The increase in the capacity may be qualitatively described in terms of a two-phase composite model with the interface layer of a high capacity. In addition, LTC composites have a better cycling rate and stability compared to LTO. This effect is caused by a higher ionic conductivity of the composites, as demonstrated in the impedance measurements above. Due to a noticeable difference in crystal structures of LTO and TiO_2_, no strong interface interaction occurs in LTT composites.

Both effects are closely interrelated, and their primary reason seems to be the influence of interfaces on the physical properties of the components of the composite, namely, on its transport properties and the electrochemical capacitance. This should be common and may be observed in different composite electrode materials containing components with similar crystal structures.

## Figures and Tables

**Table 1:** The calculated values of relative concentrations of components in composites.

Sample	Atomic Ratio Li:Ti	Mass Fraction of TiO_2_ or Li_2_TiO_3_	Volume Fraction of TiO_2_ or Li_2_TiO_3_
LTT-3	2.8:5	0.272	0.235
LTT-2	3.2:5	0.179	0.152
LTT-1	3.6:5	0.088	0.074
LTO	4:5	0	0
LTC-05	4.2:5	0.040	0.032
LTC-1	4.4:5	0.079	0.064
LTC-15	4.6:5	0.117	0.097
LTC-2	4.8:5	0.155	0.129
LTC-3	5.2:5	0.230	0.194

**Table 2:** Numerical values of the equivalent circuit parameters obtained by fitting.

Impedance	Parameters of the Equivalent Circuit	Sample					
		LTO	LTC-1	LTC-2	LTC-3	LTT-1	LTT-3
Z_b_	A_b_, S·K·cm^−1^	7 × 10^2^	2 × 10^3^	2 × 10^3^	6 × 10^3^	0.5 × 10^3^	1.2 × 10^3^
	E_b_,eV	0.47	0.48	0.48	0.53	0.48	0.48
	CPE_b_, S·cm^−1^·(Hz)^α^_b_	3 × 10^−11^	9 × 10^−10^	5 × 10^−10^	1 × 10^−11^	4 × 10^−12^	1.5 × 10^−10^
	α_b_	0.6	0.5	0.4	0.6	0.65	0.5
Z_gb_	A_gb_, S·K·cm^−1^	2.0 × 10^5^	5.7 × 10^6^	5.6 × 10^6^	3.3 × 10^7^	1.6 × 10^6^	3.7 × 10^6^
	E_gb_, eV	0.97	0.90	0.90	1.02	0.98	0.91
	CPE_gb_, S·cm^−1^·(Hz)^α^_gb_	3 × 10^−6^	1.2 × 10^−4^	2.4 × 10^−4^	9 × 10^−7^	2.2 × 10^−5^	8 × 10^−4^
	α_gb_	0.85	0.77	0.75	0.91	0.80	0.71
Z_e_	CPE_e_, S·cm^−1^·(Hz)^α^_gb_	4 × 10^−8^	8 × 10^−7^	8 × 10^−7^	3.5 × 10^−7^	1.1 × 10^−7^	5 × 10^−7^
	α_e_	0.5	0.5	0.5	0.5	0.5	0.5
σ_b_at 25 °C, S·cm^−1^		3 × 10^−8^	5 × 10^−8^	5 × 10^−8^	2 × 10^−8^	4 × 10^−8^	1 × 10^−8^
σ_dc_ at 25 °C, S·cm^−1^ *		2 × 10^−13^	1 × 10^−11^	1 × 10^−11^	1 × 10^−13^	6 × 10^−12^	5 × 10^−13^

* Calculated from the Arrhenius relation.
